# Supplementary material for: Improved jellyfish gelatin quality through ultrasound-assisted salt removal and an extraction process
Source: PLoS One. 2022 Nov 2;17(11):e0276080. doi: 10.1371/journal.pone.0276080 (PMC9629545; doi:10.1371/journal.pone.0276080)
Supplement: S1 File — (DOCX) [file pone.0276080.s001.docx]

**Table S 1** Physical and chemical properties of ultrasonicated jellyfish by-products

| **Property** | **U0 (control)** | **U20** | **U40** | **U60** | **U80** | **U100** |
| --- | --- | --- | --- | --- | --- | --- |
| Salt content before ultrasound (g/100 g) ^ns^ | 1.16 ± 0.06 | 1.16 ± 0.06 | 1.16 ± 0.06 | 1.16 ± 0.06 | 1.16 ± 0.06 | 1.16 ± 0.06 |
| Salt content after ultrasound (g/100 g) | 1.16 ± 0.06^a^ | 0.937 ± 0.02 ^b^ | 0.554 ± 0.01 ^c^ | 0.054 ± 0.00 ^d^ | 0.053 ± 0.00 ^d^ | 0.00 ± 0.00 ^e^ |
| Salt reduction (%) | 0.00 ± 0.00 ^e^ | 19.25 ± 2.63 ^d^ | 52.21 ± 0.84 ^c^ | 95.34 ± 0.00 ^b^ | 95.43 ± 0.22 ^b^ | 100 ± 0.00 ^a^ |
| Weight before ultrasound (g) ^ns^ | 200.00 ± 0.00 | 200.00 ± 0.00 | 200.00 ± 0.00 | 200.00 ± 0.00 | 200.00 ± 0.00 | 200.00 ± 0.00 |
| Weight after ultrasound (g) | 200.00 ± 0.00 ^d^ | 232.00 ± 2.71 ^b^ | 240.46 ± 3.68 ^a^ | 224.15 ± 1.54 ^c^ | 117.07 ± 2.90 ^e^ | 69.74 ± 1.01 ^f^ |
| Water absorption (%) | 0.00 ± 0.00 ^d^ | 16.00 ± 1.35 ^b^ | 20.23 ± 1.84 ^a^ | 12.08 ± 0.77 ^c^ | -41.46 ± 1.45 ^e^ | -63.13 ± 0.50 ^f^ |

**Table S 2** Physical and chemical properties of ultrasonicated jellyfish by-products

| **Determination** | **U0 (control)** | **U20** | **U40** | **U60** | **U80** | **U100** |
| --- | --- | --- | --- | --- | --- | --- |
| Jellyfish flesh |  |  |  |  |  |  |
| Salt reduction (%) | 0.00 ± 0.00^e^ | 19.25 ± 2.63^d^ | 52.21 ± 0.84^c^ | 95.34 ± 0.00^b^ | 95.43 ± 0.22^b^ | 100.00 ± 0.00^a^ |
| Water absorption (%) | 0.00 ± 0.00^d^ | 16.00 ± 1.35^b^ | 20.23 ± 1.84^a^ | 12.08 ± 0.77^c^ | -41.46 ± 1.45^e^ | -63.13 ± 0.50^f^ |
| Color |  |  |  |  |  |  |
| L* | 57.13 ±0.19^d^ | 59.42 ± 1.07^b^ | 58.73±0.83^c^ | 60.60±0.60^a^ | 52.53±0.88^e^ | 47.98±0.40^f^ |
| a* | 4.96 ± 0.57^f^ | 6.50 ± 0.45^c^ | 6.17 ±0.19^d^ | 5.58 ± 0.70^e^ | 6.78 ± 0.34^b^ | 7.04 ± 0.63^a^ |
| b* | 21.06 ±1.15^e^ | 22.04 ± 0.05^d^ | 22.51 ±0.34^c^ | 19.85± 0.02^f^ | 22.76 ±0.54^b^ | 23.41±0.53^a^ |
| Appearance | 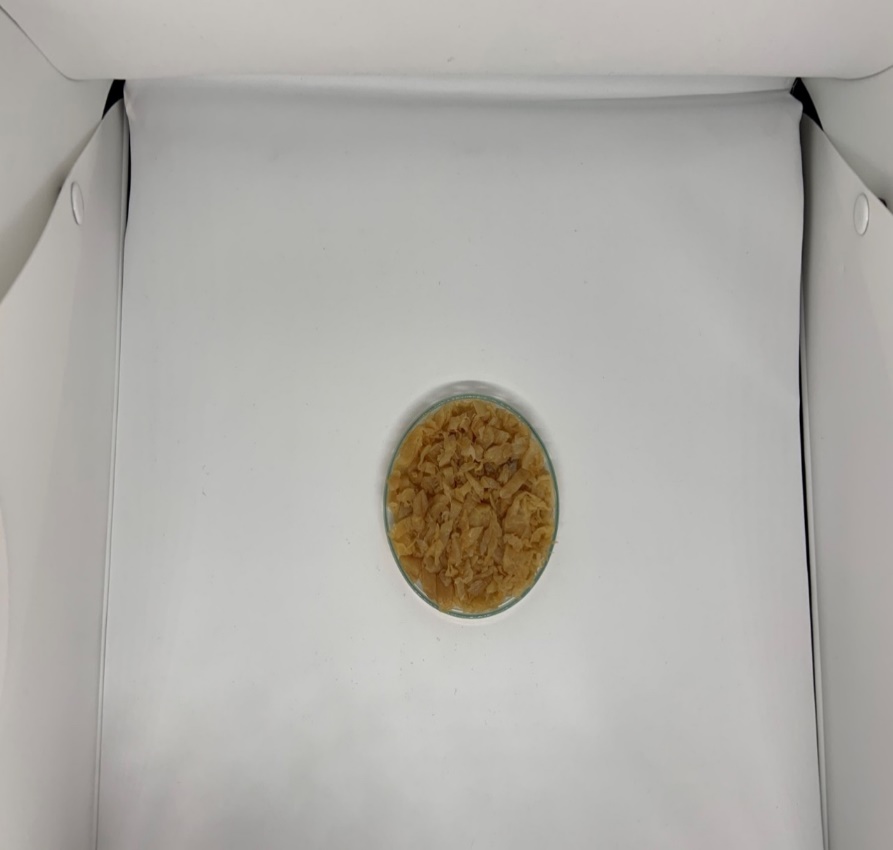 | 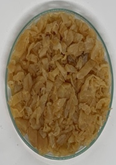 | 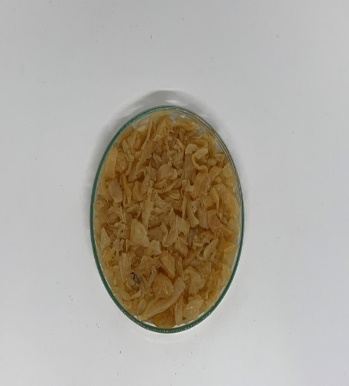 | 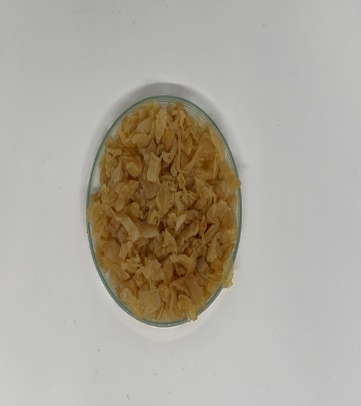 | 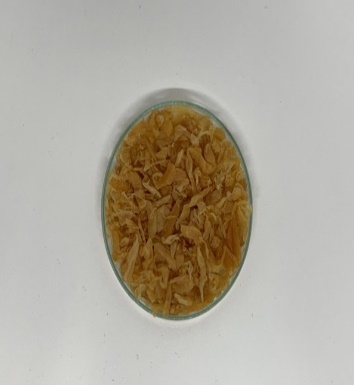 | 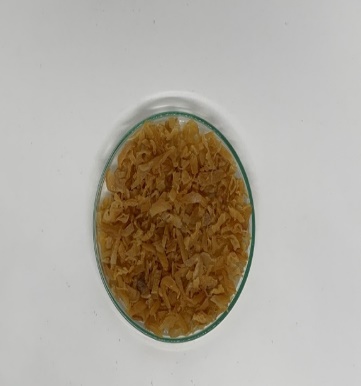 |
|  | Brown and  elastic texture | Light brown  swollen and elastic texture | Light brown swollen and elastic texture | Light brown swollen and elastic texture | Light brown swollen broken and shrunk pieces | Light brown swollen broken and shrunk pieces |
| **Water medium** |  |  |  |  |  |  |
| Conductivity^#^  (µs/cm) | 0.00 ± 0.00^f^ | 0.005± 0.88^e^ | 0.006± 0.50^d^ | 0.008± 0.30^c^ | 0.012± 0.27^b^ | 0.022 ± 0.15^a^ |
| Soluble protein^#^(mg/l) | 0.00 ± 0.00 ^e^ | 0.303± 0.27^e^ | 0.311± 0.36^d^ | 0.344± 0.79^c^ | 0.427± 0.90^b^ | 0.528 ± 0.63^a^ |
|  |  |  |  |  |  |  |
|  |  |  |  |  |  |  |

The ultrasound settings used were 40 kHz and 220 W. The duration time of ultrasound treatment was 0, 20, 40, 60, 80, and 100 min, designated as U0, U20, U40, U60, U80, and U100, respectively.

The values are the mean of triplicate ± SD. Different superscripts in the same row are significantly different (p≤0.05).

Remark: ^#^The conductivity and soluble protein properties were measured in the water medium of the sonication bath.

**Table S 3** Peak wave numbers of desalted jellyfish powder and jellyfish gelatin prepared from by-products with different ultrasonication time

| Region | Peak wave number (cm^-1^) | | | | | | |
| --- | --- | --- | --- | --- | --- | --- | --- |
|  | U0 | U20 | U40 | U60 | U80 | U100 | Assignment |
| Amide A | 3438.57 | 3400.82 | 3432.83 | 3433.65 | 3424.62 | 3436.11 | N-H stretch coupled with hydrogen bond |
| Amide B | 2931.32 | 2928.86 | 2926.40 | 2925.74 | 2923.93 | 2920.65 | CH_2_ asymmetrical stretch |
| Amide I | 1661.55 | 1659.09 | 1656.63 | 1653.35 | 1650.88 | 1639.39 | C=O stretch/hydrogen bond coupled with COO- |
| Amide II | 1550.75 | 1547.46 | 1545.00 | 1542.54 | 1539.26 | 1536.79 | NH bend coupled with CN stretch, |
| Amide III | 1244.59 | 1243.77 | 1241.31 | 1238.85 | 1236.38 | 1228.18 | NH bend coupled with CN stretch, C-O stretch |

| Region | Peak wave number (cm^-1^) | | | | | | |
| --- | --- | --- | --- | --- | --- | --- | --- |
|  | JFG0-4 | JFG20-4 | JFG40-4 | JFG60-4 | JFG80-4 | JFG100-4 | Assignment |
| Amide A | 3438.57 | 3436.36 | 3437.75 | 3435.29 | 3433.84 | 3430.36 | N-H stretch coupled with hydrogen bond |
| Amide B | 2932.94 | 2932.92 | 2931.67 | 2930.69 | 2924.85 | 2920.31 | CH_2_ asymmetrical stretch |
| Amide I | 1649.75 | 1643.29 | 1643.06 | 1636.47 | 1631.93 | 1629.65 | C=O stretch/hydrogen bond coupled with COO- |
| Amide II | 1554.03 | 1549.50 | 1545.04 | 1541.72 | 1538.76 | 1529.41 | NH bend coupled with CN stretch |
| Amide III | 1246.53 | 1245.64 | 1238.82 | 1237.62 | 1234.28 | 1229.73 | NH bend coupled with CN stretch, C-O stretch |

**Table S 3** Peak wave numbers of desalted jellyfish powder and jellyfish gelatin prepared from by-products with different ultrasonication time (Cont.)

**Table S 3** Peak wave numbers of desalted jellyfish powder and jellyfish gelatin prepared from by-products with different ultrasonication time (Cont.)

| Region | Peak wave number (cm^-1^) | | | | | | |
| --- | --- | --- | --- | --- | --- | --- | --- |
|  | JFG0-6 | JFG20-6 | JFG40-6 | JFG60-6 | JFG80-6 | JFG100-6 | Assignment |
| Amide A | 3439.86 | 3439.23 | 3438.67 | 3434.69 | 3431.54 | 3426.86 | N-H stretch coupled with hydrogen bond |
| Amide B | 2892.02 | 2969.19 | 2961.80 | 2962.13 | 2969.30 | 2944.25 | CH_2_ asymmetrical stretch |
| Amide I | 1688.00 | 1687.95 | 1687.37 | 1687.56 | 1688.42 | 1688.70 | C=O stretch/hydrogen bond coupled with COO- |
| Amide II | 1525.09 | 1525.14 | 1525.35 | 1525.53 | 1525.40 | 1526.11 | NH bend coupled with CN stretch |
| Amide III | 1254.12 | 1244.38 | 1243.91 | 1244.55 | 1245.64 | 1245.07 | NH bend coupled with CN stretch, C-O stretch |

**Table S 3**  Peak wave numbers of desalted jellyfish powder and jellyfish gelatin prepared from by-products with different ultrasonication time (Cont.)

| Region | Peak wave number (cm^-1^) | | | | | | |
| --- | --- | --- | --- | --- | --- | --- | --- |
|  | JFG0-8 | JFG20-8 | JFG40-8 | JFG60-8 | JFG80-8 | JFG100-8 | Assignment |
| Amide A | 3417.86 | 3423.14 | 3415.87 | 3415.26 | 3414.89 | 3410.70 | N-H stretch coupled with hydrogen bond |
| Amide B | 2931.60 | 2971.83 | 2973.37 | 2967.75 | 2968.17 | 2967.72 | CH_2_ asymmetrical stretch |
| Amide I | 1644.82 | 1644.71 | 1689.82 | 1688.85 | 1682.33 | 1687.80 | C=O stretch/hydrogen bond coupled with COO- |
| Amide II | 1534.83 | 1534.61 | 1550.82 | 1550.43 | 1534.59 | 1525.27 | NH bend coupled with CN stretch |
| Amide III | 1243.99 | 1288.67 | 1285.56 | 1251.09 | 1245.24 | 1243.44 | NH bend coupled with CN stretch, C-O stretch |

**Table S 4** Yield (%) and gel strength (g) of jellyfish gelatin prepared from by-products with different ultrasonication time

| **Sample** | **Yield (%)** | **Gel strength (g)** |
| --- | --- | --- |
| JFG0-4 | 7.43±0.15 ^m^ | 129.62±1.10 ^k^ |
| JFG20-4 | 8.81±0.27 ^l^ | 160.05±1.63 ^i^ |
| JFG40-4 | 10.60±0.56 ^j^ | 447.01±1.06 ^a^ |
| JFG60-4 | 12.30±0.72 ^j^ | 283.29±2.36 ^b^ |
| JFG80-4 | 16.21±0.30 ^i^ | 278.71±2.90 ^c^ |
| JFG100-4 | 17.15±0.83 ^h^ | 121.66±2.27 ^l^ |
| JFG0-6 | 9.05±0.15 ^k^ | 188.70±1.79 ^g^ |
| JFG20-6 | 12.31±0.30^j^ | 246.76±1.98 ^d^ |
| JFG40-6 | 12.11±0.53 ^j^ | 278.91±1.59 ^c^ |
| JFG60-6 | 12.31±0.72 ^j^ | 191.98±3.04 ^f^ |
| JFG80-6 | 16.49±0.56 ^i^ | 150.33±2.95 ^j^ |
| JFG100-6 | 18.57±0.88 ^g^ | 120.06±1.10 ^l^ |
| JFG0-8 | 26.87±0.85 ^e^ | 248.12±3.60 ^d^ |
| JFG20-8 | 27.04±0.96 ^d^ | 207.75±2.55 ^e^ |
| JFG40-8 | 29.29±1.23 ^c^ | 181.57±1.42 ^h^ |
| JFG60-8 | 31.77±1.27 ^b^ | 152.95±2.88 ^j^ |
| JFG80-8 | 32.69±1.98 ^a^ | 114.92±1.85^m^ |
| JFG100-8 | 25.77±0.94^f^ | 91.89±1.74^n^ |
