# Supplementary material for: Improved jellyfish gelatin quality through ultrasound-assisted salt removal and an extraction process
Source: PLoS One. 2022 Nov 2;17(11):e0276080. doi: 10.1371/journal.pone.0276080 (PMC9629545; doi:10.1371/journal.pone.0276080)
Supplement: Attachment — Submitted filename: Response to Reviewers.docx [file pone.0276080.s003.docx]

Dear Academic Editor and Reviewers,

Below are the responses to the academic editor and reviewers' comments. All answers are in red.

**Journal Requirements:**

Please review your reference list to ensure that it is complete and correct. If you have cited papers that have been retracted, please include the rationale for doing so in the manuscript text, or remove these references and replace them with relevant current references. Any changes to the reference list should be mentioned in the rebuttal letter that accompanies your revised manuscript. If you need to cite a retracted article, indicate the article’s retracted status in the References list and also include a citation and full reference for the retraction notice.

Ans. The reference has been checked to complete and correct.

Reviewer #2: Review PONE-D-22-06129

This manuscript is improved and the authors answer my questions properly. However, I have a quick question why the data on salt reduction in Table 1 of revised manuscript are different from the original manuscript, please explain. Some information should be added in the manuscript. For example, the ambient temperature should be indicated in the manuscript so the readers will understand the work better.

Ans : The data in Table 1 of revised manuscript has been recalculated using the equation in Line 175-179, including
Eq.(1) is salt content (%) = 5.8 x (V_1_ x N_1_) – (V_2_ x N_2_/ W)
Eq.(2) is salt reduction (%) = (S_0_-S_i_)/S_0_*100

In this case, the salt content of each condition (U0, U20, U40, U60, U80, and U100) was initially measured. After calculation by using Eq. (2), salt reduction with respect to the control displayed the clear reducing trend of salt content. This is quite different from the previous data in original manuscript that reported the increased or decreased weight (g) of the sample after sonication. These raw data were hardly to explain the effect of sonication on salt content. Therefore, the additional sentence has been added in Line 298-300 that is “The salt content of each condition (U0, U20, U40, U60, U80 and U100) was initially measured ” (Table S1), and the salt reduction compared to the control (U0) was then calculated”.

The temperature of the water medium was 25 ± 3^o^C (ambient temperature) has been added in Line 311.

If you have any concerns, please feel free to contact me.

Best regards,

Benjawan Thumthanaruk

Corresponding author
